# How Is Bullying Victimization Associated with Depressive Symptoms Among Multicultural Adolescents in South Korea? A Serial Mediation and Network Analysis of Acculturative Stress and Ego-Resiliency

**DOI:** 10.3390/bs16091667

**Published:** 2026-09-16

**Authors:** Zhenping Jiang, Xiaochen Li, Bum-Young Park, Meng Chen

**Affiliations:** Sport Psychology Lab, Department of Sports Science, Hanyang University ERICA, 55 Hanyangdaehak-ro, Sangnok-gu, Ansan-si 15588, Gyeonggi-do, Republic of Korea; jiangzhenping333@gmail.com (Z.J.); hyby68@hanyang.ac.kr (B.-Y.P.); chenm4512@hanyang.ac.kr (M.C.)

**Keywords:** bullying victimization, depressive symptoms, acculturative stress, ego-resiliency, multicultural adolescents, network analysis

## Abstract

Bullying victimization is consistently associated with depressive symptoms among adolescents, yet the psychological processes underlying this association remain insufficiently understood, particularly among multicultural adolescents. This study tested a theoretically specified serial mediation model involving acculturative stress and ego-resiliency and further explored item-level conditional associations among the study variables. Cross-sectional data were obtained from 1338 multicultural adolescents in South Korea who participated in the fifth wave (2015) of the Multicultural Adolescents Panel Study (MAPS). Serial mediation analysis (PROCESS Model 6) was performed to examine the mediating roles of acculturative stress and ego-resiliency. A Gaussian graphical network was subsequently estimated to identify central nodes, bridge nodes, and node predictability among bullying victimization, acculturative stress, ego-resiliency, and depressive symptoms. Bullying victimization was positively associated with depressive symptoms. Significant indirect associations were observed through acculturative stress and ego-resiliency, both independently and through the theoretically specified serial pathway. In the network, physical aggression (BV4), appearance-related humiliation (BV6), and peer exclusion (BV1) occupied relatively central positions. Rumor spreading (BV5) showed the highest bridge expected influence, whereas BV4 and BV6 showed the highest bridge strength. Acculturative-stress items demonstrated the highest average node predictability. Bullying victimization was associated with depressive symptoms among multicultural adolescents in South Korea, with significant indirect associations involving greater acculturative stress and lower ego-resiliency. Serial mediation and network analyses provided complementary construct- and item-level perspectives, but the cross-sectional findings should be interpreted as associational rather than causal.

## 1. Introduction

In recent years, the increasing number of transnational marriages has gradually transformed South Korea from a traditionally ethnically homogeneous society into a multicultural society (Jang et al., 2024). Multicultural families generally refer to families whose members have different nationalities or cultural backgrounds (Chung & Jung, 2012). According to statistics released by the Korean Ministry of Gender Equality and Family, the number of multicultural families in South Korea increased from approximately 299,000 in 2015 to 439,300 in 2024. During the same period, the population of multicultural adolescents aged 9–24 years increased markedly from approximately 55,000 in 2013 to 202,000 in 2025 (Ministry of Gender Equality and Family, 2025). As the number of multicultural adolescents continues to increase, understanding the mental health challenges experienced by this population has become increasingly important for the development of school-based and community-based support in South Korea. However, compared with their non-multicultural peers, these adolescents are more likely to experience psychological difficulties due to challenges related to cultural adaptation, identity conflicts, and social exclusion (Bae, 2020; Bahk et al., 2017; Lipsicas & Mäkinen, 2010). Therefore, identifying the factors associated with the mental health of multicultural adolescents is of considerable importance for promoting their healthy development and informing targeted psychological interventions.

Bullying is characterized by intentional and repeated aggressive behaviors that occur within a context of power imbalance among peers (Olweus, 2013), and it typically includes verbal, physical, and relational forms of victimization (Crick & Grotpeter, 1995). International surveys have reported that approximately 30.5% of adolescents experienced bullying on at least one day during the previous 30 days (Biswas et al., 2020). Similarly, a cross-national study involving adolescents from 29 countries found that the average prevalence of bullying victimization reached 39.4% (Smith et al., 2023). Owing to differences in cultural background, physical appearance, and language, multicultural adolescents often constitute a minority group within school settings and are consequently more vulnerable to peer victimization than their non-multicultural peers (Park et al., 2024). Previous studies have shown that bullying victimization not only undermines adolescents’ social support and coping capacity (Sahle et al., 2022) but also exerts persistent adverse effects on mental health, with depressive symptoms representing one of the most common and severe psychological consequences (Hawker & Boulton, 2000). According to the Interpersonal Risk Model, negative interpersonal experiences, such as bullying victimization, constitute important risk factors for emotional distress and psychological problems during adolescence (Hammen, 1992). Empirical evidence has consistently demonstrated that adolescents who experience bullying victimization exhibit higher levels of depressive symptoms, and that the risk of developing depression in adulthood increases with the frequency of bullying experiences (Ye et al., 2023). Furthermore, longitudinal studies have shown that persistent bullying victimization and the resulting depressive symptoms, if left unaddressed, may lead to a wide range of long-term adverse outcomes, including poor mental and physical health, suicidal behaviors, delinquency, and impaired socioeconomic functioning (Arseneault, 2018; Klomek et al., 2015). Collectively, these findings indicate that bullying victimization is a major risk factor for depressive symptoms among adolescents. Nevertheless, although the association between bullying victimization and depressive symptoms has been well established, the psychological mechanisms underlying this relationship remain insufficiently understood.

Beyond its direct association with depressive symptoms, bullying victimization may also influence adolescents’ mental health through multiple psychosocial pathways. One potential mechanism is acculturative stress, which refers to the psychological stress experienced during the process of adapting to a new cultural environment as a result of language barriers, cultural value conflicts, perceived discrimination, and social exclusion (Berry et al., 1987). Acculturation theory suggests that adolescents from multicultural backgrounds often encounter additional challenges during the process of adapting to the mainstream culture, placing them at increased risk of experiencing acculturative stress (Berry, 2005). In particular, multicultural adolescents may experience elevated levels of acculturative stress because of discrepancies between the cultural values of their foreign-born parents and those of the broader Korean society (Ward & Kennedy, 1993). Experiences of bullying victimization may further intensify feelings of cultural conflict and social exclusion, thereby exacerbating acculturative stress (Son et al., 2024). Accumulating evidence has identified acculturative stress as an important risk factor for depressive symptoms among multicultural adolescents. Previous studies have consistently reported that higher levels of acculturative stress are associated with more severe depressive symptoms among immigrant populations (González et al., 2001; Mui & Kang, 2006). More recently, research involving Korean multicultural adolescents has likewise demonstrated a close association between increased acculturative stress and elevated depressive symptoms (Bae, 2020). Taken together, these findings suggest that acculturative stress may represent an important psychological pathway linking bullying victimization to depressive symptoms.

In addition to acculturative stress, ego-resiliency may represent another psychological resource relevant to the association between bullying victimization and depressive symptoms. Ego-resiliency refers to an individual’s capacity to flexibly adapt to changing environmental demands and to effectively regulate emotions and behaviors when confronted with stress or adversity (Davydov et al., 2010; Rutter, 2006). Adolescents with higher ego-resiliency generally report better adaptation to negative life events and psychological well-being (Sarubin et al., 2015; Troy et al., 2023), whereas lower ego-resiliency has been associated with greater emotional and behavioral difficulties, including depressive symptoms (Liu et al., 2015). Conservation of Resources theory proposes that stressful experiences may be accompanied by the loss or reduced availability of psychological resources (Min et al., 2015). From this perspective, bullying victimization may be associated with lower ego-resiliency (Yu, 2020), and persistent acculturative stress may similarly co-occur with fewer psychological resources available for adaptation to adversity (Hu et al., 2015). Previous studies have also found that higher ego-resiliency is associated with fewer depressive symptoms among adolescents (Zhou et al., 2017), whereas lower ego-resiliency is associated with more severe depressive symptoms (E. E. Jones et al., 2023; Ning et al., 2025). These findings support the theoretical relevance of ego-resiliency to the associations among bullying victimization, acculturative stress, and depressive symptoms.

The proposed mediator ordering was theoretically based on the distinction between sociocultural stress appraisal and psychological resources for adaptation. Bullying victimization may be experienced by multicultural adolescents as cultural rejection and may therefore be associated with greater acculturative stress (Berry, 2005; Son et al., 2024). Conservation of Resources and resilience perspectives further suggest that sustained stress may be associated with the reduced availability or flexibility of psychological resources, supporting the theoretical placement of acculturative stress before ego-resiliency (Min et al., 2015; Rutter, 2006). However, this ordering is theoretical rather than temporal, and the reverse ordering cannot be excluded using cross-sectional data. The joint consideration of acculturative stress and ego-resiliency is particularly relevant to multicultural adolescents in South Korea. Acculturative stress reflects challenges associated with navigating the culture of a foreign-born parent and the dominant Korean culture, including those related to language, appearance, family background, and social acceptance (Bae, 2020; Ward & Kennedy, 1993). Although ego-resiliency is not culturally specific in itself, it may represent an important adaptive resource for adolescents managing both normative developmental demands and stress associated with bicultural adaptation and minority status (Davydov et al., 2010; Rutter, 2006; Yu, 2020). Examining these factors together therefore provides a culturally relevant framework for understanding the association between bullying victimization and depressive symptoms in this population.

Despite the growing body of research on bullying victimization and adolescent mental health, several important gaps remain. First, relatively few studies have focused specifically on multicultural adolescents in South Korea, despite evidence suggesting that this population may face unique mental health risks because of cultural background differences, identity conflicts, and challenges in social adaptation. Second, previous studies have primarily examined the mediating roles of acculturative stress or ego-resiliency separately, whereas evidence regarding their theoretically specified serial pattern of associations remains limited. Examining this theoretically proposed pattern may provide a more comprehensive understanding of the association between bullying victimization and depressive symptoms among multicultural adolescents in South Korea. In addition, existing studies have predominantly adopted variable-centered approaches, such as regression analyses and mediation models, to examine overall relationships among psychological constructs. Although these approaches are valuable for testing theoretical associations, they provide limited insight into the complex interactions among individual psychological experiences and their respective roles within the broader psychological system.

Network analysis has emerged as a valuable analytical framework for examining psychological phenomena at the item level by conceptualizing individual symptoms or psychological components as an interconnected system rather than focusing solely on associations among aggregate scale scores (Epskamp et al., 2016; Fried et al., 2017). Within a symptom network, nodes represent individual symptoms or components, whereas edges represent conditional associations between them after controlling for all other nodes in the network (Borsboom, 2017). Although individual partial correlations can describe selected conditional associations, they do not provide an integrated representation of the overall multivariate structure across all items. By estimating the network as a whole, this approach enables the characterization of within- and between-community connections and the identification of highly connected nodes, bridge nodes, and node predictability, which reflects the proportion of variance in each node explained by its neighboring nodes (Haslbeck & Fried, 2017; P. J. Jones et al., 2021). Compared with traditional mediation analysis, network analysis provides a complementary perspective by revealing heterogeneous item-level conditional associations that may be obscured when only scale-level scores are examined (Fried & Cramer, 2017). Accordingly, in the present study, network analysis was used as an exploratory and complementary approach to describe item-level associations among bullying victimization, acculturative stress, ego-resiliency, and depressive symptoms; it was not intended to validate the serial mediation model or establish causal pathways. To our knowledge, few studies have simultaneously examined these associations using both serial mediation and network analysis among multicultural adolescents in South Korea.

### The Present Study

**Figure 1:**
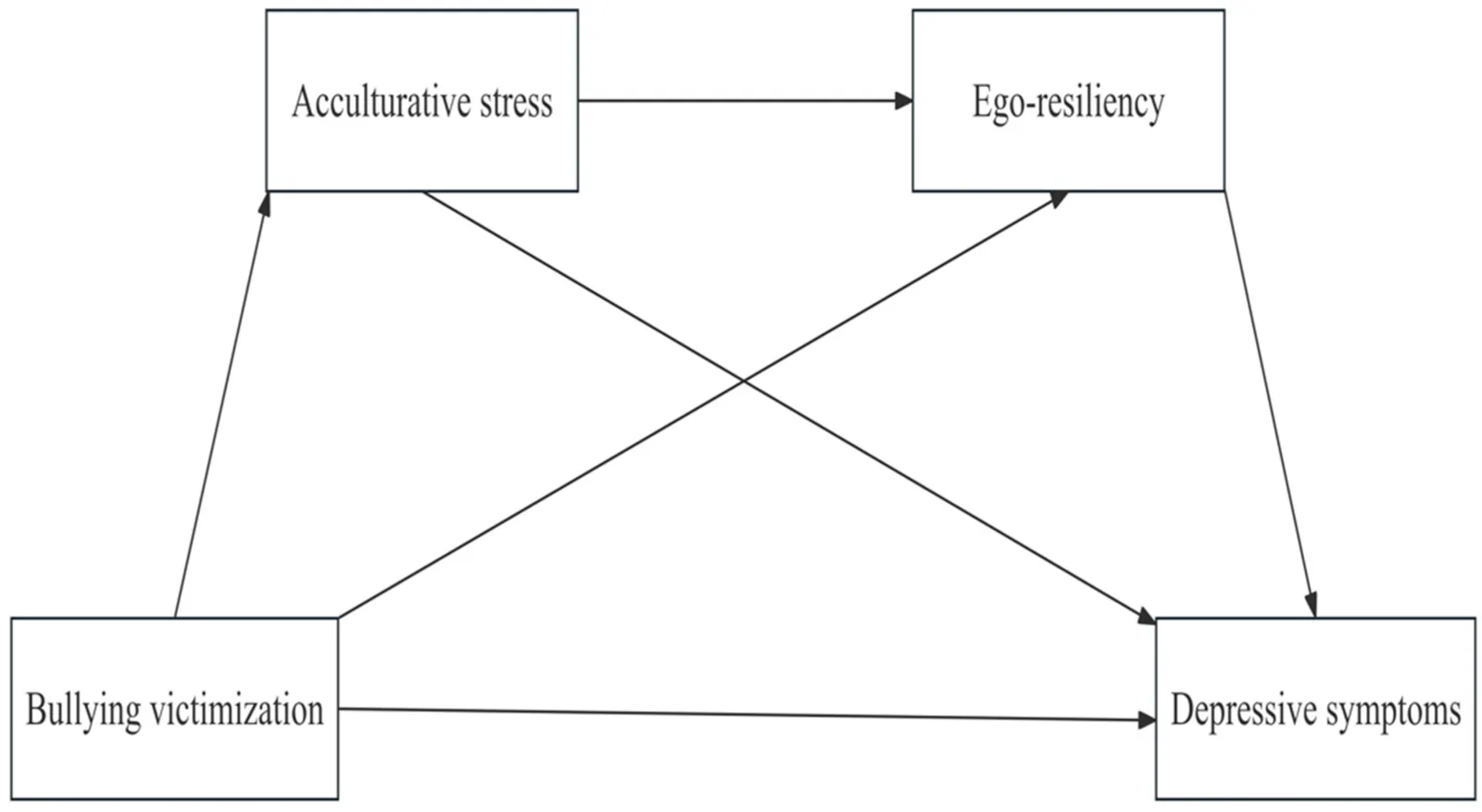
The hypothetical serial mediation model of acculturative stress and ego-resiliency in the association between bullying victimization and depressive symptoms among Korean multicultural adolescents.

The present cross-sectional study examined associations among bullying victimization, acculturative stress, ego-resiliency, and depressive symptoms among Korean multicultural adolescents. Guided by acculturation and resilience perspectives, we evaluated whether the observed associations were consistent with a theoretically proposed serial mediation model in which acculturative stress and ego-resiliency were specified as sequential mediators of the association between bullying victimization and depressive symptoms. Because all variables were assessed at one time point, this model was not intended to establish temporal or causal ordering. We also conducted an exploratory item-level network analysis to characterize conditional associations among items and to identify nodes with high centrality, bridge centrality, and predictability. The proposed model is presented in Figure 1.

**Hypothesis** **1.** *Bullying victimization would be positively associated with depressive symptoms among Korean multicultural adolescents.*

**Hypothesis** **2.** *Acculturative stress and ego-resiliency would serially mediate the association between bullying victimization and depressive symptoms.*

Given the exploratory nature of network analysis, no explicit hypothesis was formulated regarding the symptom-level relationships among bullying victimization, acculturative stress, ego-resiliency, and depressive symptoms.

## 2. Methods

### 2.1. Study Procedure and Participants

This study used data from the Multicultural Adolescents Panel Study (MAPS), conducted by the National Youth Policy Institute (NYPI) of South Korea. MAPS is a nationwide longitudinal panel study designed to systematically collect information on the sociocultural experiences, mental health, family characteristics, and social adjustment of children and adolescents from multicultural families. At baseline in 2011, MAPS recruited 1635 fourth-grade elementary school students from multicultural families, aged approximately 9–10 years, together with their mothers. This sample accounted for approximately 36–37% of all fourth-grade multicultural students in South Korea at that time. A two-stage stratified sampling procedure was employed. First, schools were stratified according to the regional distribution of multicultural students across 16 metropolitan cities and provinces in South Korea and were then randomly selected. Second, probability proportional-to-size sampling was applied to increase the likelihood of selecting schools with larger numbers of multicultural students. Ultimately, 1635 adolescents were selected from a sampling frame comprising 4452 multicultural students attending 2537 elementary schools nationwide. Data collection commenced in 2011, and participants were subsequently followed annually. Trained interviewers collected the data through face-to-face household interviews. Before participation, parents were informed of the study purpose and procedures, and written informed consent was obtained. To facilitate participation among multicultural families, the parent questionnaire was available in 10 languages: Korean, English, simplified Chinese, traditional Chinese, Japanese, Filipino, Vietnamese, Mongolian, Russian, and Thai. The adolescent questionnaire was administered in Korean.

The present study used data from the fifth main wave of MAPS, collected in 2015. Wave 5 was selected because it was the most recent MAPS wave in which all four focal measures, including ego-resiliency, were administered concurrently. Of the 1635 adolescents enrolled at baseline, 1347 participated in Wave 5, corresponding to a retention rate of 82.4% and an attrition rate of 17.6%. To ensure that the analytic sample met the definition of a multicultural family, one participant whose parents were both born in South Korea was excluded. A further eight participants with missing data on perceived family economic status were excluded. The final analytic sample therefore comprised 1338 adolescents. Because only eight Wave 5 participants had missing data on the study variables (0.59%), complete-case analysis using listwise deletion was applied (Allison, 2024).

The MAPS protocol was reviewed and approved through the relevant ethical procedures of the National Youth Policy Institute, and informed consent was obtained from all participants and their legal guardians before data collection. The present study used only publicly available, anonymized secondary data and involved neither personal identification nor additional data collection; therefore, no additional ethical approval was required.

### 2.2. Measures

#### 2.2.1. Bullying Victimization

Bullying victimization was assessed using six items adapted from Lee and Kim (Lee & Kim, 2001). Participants were asked how frequently they had experienced different forms of bullying by other students during the current semester. The items assessed the following experiences: (1) being excluded by other students; (2) being insulted, bullied, or mocked; (3) being deliberately ignored; (4) being physically attacked, such as being hit, kicked, or threatened; (5) being disliked by friends because of rumors spread by other students; and (6) being harassed or mocked because of physical characteristics or appearance. Responses were rated on a four-point scale ranging from 1 (never) to 4 (almost every day), with higher summed scores indicating more frequent bullying-victimization experiences. The same six-item MAPS measure has previously been operationalized as bullying victimization among multicultural adolescents in South Korea (Park et al., 2024). Although the response categories capture the frequency of victimization during the current semester, the items do not directly assess a power imbalance between the perpetrator and the victim. Therefore, bullying victimization in the present study should be understood as a relatively broad operationalization encompassing multiple forms of peer victimization rather than as a strict assessment of all conventional defining criteria for bullying. The scale demonstrated excellent internal consistency in the present study (Cronbach’s α = 0.907).

#### 2.2.2. Depressive Symptoms

Depressive symptoms were assessed using a 10-item adolescent adaptation of the depression subscale of the Symptom Checklist-90-Revised (SCL-90-R). The original scale was developed by Derogatis et al. and subsequently standardized for use in South Korea (Kim et al., 1978). The 10-item version was derived from the 13-item Korean SCL-90-R depression subscale by omitting three items and adapting the retained items for administration to adolescents. The items assessed depressive experiences including sadness, loneliness, hopelessness about the future, lack of energy, self-blame, and loss of interest in activities. Each item was rated on a four-point Likert scale ranging from 1 (strongly disagree) to 4 (strongly agree), with higher summed scores indicating more severe depressive symptoms. In the present sample, exploratory factor analysis using principal axis factoring supported a single-factor structure (KMO = 0.920; Bartlett’s χ^2^(45) = 7421.009, *p* < 0.001). The factor accounted for 51.1% of the variance, and factor loadings ranged from 0.578 to 0.825. The scale demonstrated excellent internal consistency (Cronbach’s α = 0.907).

#### 2.2.3. Acculturative Stress

Acculturative stress was assessed using the 10-item measure included in the Multicultural Adolescents Panel Study. This measure was based on the Social, Attitudinal, Familial, and Environmental Acculturative Stress Scale developed by Hovey and King and subsequently translated and adapted for use in South Korea by Nho and Hong (Hovey & King, 1996; Nho & Hong, 2006), as described in previous research using MAPS data. A previous study using the same MAPS measure excluded one item because of a low item–total correlation and used the remaining nine items in the analysis (Bae, 2020). In the present sample, Item 10 (“I would be able to live better in Korea than in my foreign-born parent’s country”) also showed poor psychometric performance, with a corrected item–total correlation of 0.002 and an extraction communality of 0.037. The item failed to show a salient factor loading of 0.30 or higher and was therefore removed. Following its removal, Cronbach’s alpha increased from 0.763 to 0.851.

#### 2.2.4. Ego-Resiliency

Ego-resiliency was assessed using the 14-item Ego-Resiliency Scale developed by (Block & Kremen, 1996). The scale measures adolescents’ adaptive capacity, interpersonal openness, and psychological flexibility. Each item was rated on a four-point Likert scale ranging from 1 (strongly disagree) to 4 (strongly agree), with higher scores indicating greater ego-resiliency. The scale demonstrated excellent internal consistency in the present study (Cronbach’s α = 0.910).

#### 2.2.5. Covariates

Previous research has shown that depressive symptoms and experiences of bullying victimization may vary according to adolescents’ sex, age, and socioeconomic circumstances (Li et al., 2025; Park et al., 2024). In particular, sex and age are commonly associated with variations in emotional symptoms and peer experiences during adolescence, while lower perceived family economic status has been linked to greater psychosocial stress and poorer mental health outcomes. Therefore, adolescent sex, age, and perceived family economic status were included as covariates in the serial mediation model. Sex was coded as 1 for male and 2 for female. Age was treated as a continuous variable and expressed in years. Perceived family economic status was assessed using five response categories: very poor, poor, average, good, and very good. Higher scores indicated a more favorable perception of family economic status.

#### 2.2.6. Statistical Analysis

All analyses were conducted using IBM SPSS Statistics version 25 and R version 4.5.2.

Descriptive statistics were first calculated to summarize the participants’ sociodemographic characteristics, including age, sex, parental country-of-birth status, and perceived family economic status. Pearson correlation coefficients were subsequently calculated to examine the bivariate associations among bullying victimization, acculturative stress, ego-resiliency, and depressive symptoms. To test the proposed hypotheses, serial mediation analysis was conducted using Hayes’ PROCESS macro, version 4.1, Model 6. Bullying victimization was specified as the independent variable (X), depressive symptoms as the dependent variable (Y), acculturative stress as the first mediator (M1), and ego-resiliency as the second mediator (M2). Age, sex, and perceived family economic status were entered as covariates. Percentile bootstrap confidence intervals based on 5000 resamples were used to estimate the total indirect effect and each specific indirect effect. An indirect effect was considered statistically significant when its 95% bootstrap confidence interval did not include zero. Unstandardized regression coefficients (B), standard errors (SEs), *p* values, and 95% confidence intervals were reported for the regression analyses, whereas standardized path coefficients (β) were presented in the serial mediation model figure. As a sensitivity analysis, an alternative Model 6 specifying ego-resiliency before acculturative stress was estimated using the same covariates and 5000 percentile bootstrap samples. All analyses were conducted without applying survey weights or design-based adjustments for stratification and clustering.

To further investigate item-level associations among bullying victimization, acculturative stress, ego-resiliency, and depressive symptoms, a network analysis was conducted. This item-level network analysis was intended to complement, rather than validate, the construct-level serial mediation model. Each scale item was represented as a node, and edges represented regularized partial correlations between pairs of nodes after controlling for all other nodes in the network. Because all items were measured using four-point ordinal response scales, polychoric correlations were estimated for all item pairs using the cor_auto() function (Epskamp et al., 2018). The Gaussian graphical model was applied to the resulting polychoric correlation matrix within a latent-response framework, assuming that the observed ordinal responses reflected underlying continuous variables.

The network was estimated as a Gaussian graphical model using the extended Bayesian information criterion graphical least absolute shrinkage and selection operator (EBICglasso) procedure, with the EBIC hyperparameter set to γ = 0.50 (Friedman et al., 2008). The network was visualized using the Fruchterman–Reingold spring layout. The 39 nodes were assigned a priori to four theoretically defined communities according to their respective measurement scales: bullying victimization (6 items), acculturative stress (9 items), ego-resiliency (14 items), and depressive symptoms (10 items). No missing item-level responses remained in the final analytic dataset; therefore, no additional imputation or item-level missing-data procedure was required for the network analysis.

Node centrality was assessed using strength, defined as the sum of the absolute edge weights connected to a node, and expected influence, defined as the sum of the corresponding signed edge weights (Fried et al., 2017). Predictability represented the proportion of variance in each node accounted for by its neighboring nodes. Bridge strength and one-step bridge expected influence were calculated to assess absolute and signed cross-community connectivity, respectively (Heeren et al., 2018). In practical terms, a highly connected node is an item that shows relatively strong conditional associations with many other items in the network, whereas a bridge node is an item that connects its own theoretical community with items belonging to other communities (Fried & Cramer, 2017; Heeren et al., 2018). These indices reflect statistical connectivity within the estimated network and should not be interpreted as evidence of causal or clinical importance. Closeness and betweenness were included only in the stability assessment and were not used for substantive interpretation because of their limitations in regularized psychological networks.

Potential node redundancy was examined using the Goldbricker procedure. Pairs were considered potentially redundant when they had a zero-order correlation of at least 0.50 and fewer than 25% of their correlations with the remaining nodes differed significantly at *p* < 0.01. All nodes were retained to preserve the original scale structure and content coverage.

The accuracy of the edge-weight estimates was assessed using 1000 nonparametric bootstrap samples. The stability of node centrality was examined using 2000 case-dropping bootstrap samples, and the correlation stability coefficient was calculated (Mullarkey et al., 2019). Because no single conventional closed-form power calculation is available for an EBICglasso network of this type, the adequacy of the network estimation was evaluated empirically using these nonparametric and case-dropping bootstrap procedures. In accordance with previous recommendations, a correlation stability coefficient below 0.25 was considered inadequate, whereas a coefficient of 0.50 or higher indicated good centrality stability (Epskamp et al., 2016). The corresponding stability results are reported in Section 3.4.4.

Network estimation and visualization were performed using the qgraph package (version 1.10.1), bootstrap analyses using the bootnet package (version 1.9.1), node predictability using the mgm package (version 1.2.15), and bridge-centrality and node-redundancy analyses using the networktools package (version 1.6.0).

## 3. Results

### 3.1. Sociodemographic Characteristics

Table 1 presents the descriptive characteristics of the study sample. The final analytic sample comprised 1338 adolescents from multicultural families, with a mean age of 13.97 years (SD = 0.37). The sex distribution was relatively balanced, with 657 boys (49.1%) and 681 girls (50.9%). Most participants had a foreign-born mother (96.4%), whereas 3.4% had a foreign-born father and 0.2% had two foreign-born parents. The most frequently reported maternal countries of birth were Japan (34.2%), the Philippines (25.4%), and China among ethnic Koreans (18.5%). Most adolescents perceived their household economic status as average (42.5%) or poor (40.6%). Regarding place of residence, 44.8% lived in small cities, followed by rural areas (28.8%) and large cities (26.5%).

### 3.2. Descriptive Statistics and Correlation Analysis

Table 2 presents the means, standard deviations, and Pearson correlation coefficients for the study variables. Bullying victimization was significantly and positively correlated with acculturative stress (r = 0.194, *p* < 0.01) and depressive symptoms (r = 0.204, *p* < 0.01), and significantly and negatively correlated with ego-resiliency (r = −0.135, *p* < 0.01). Acculturative stress was significantly and negatively correlated with ego-resiliency (r = −0.241, *p* < 0.01) and significantly and positively correlated with depressive symptoms (r = 0.357, *p* < 0.01). In addition, ego-resiliency was significantly and negatively correlated with depressive symptoms (r = −0.464, *p* < 0.01). Overall, the directions of the correlations among the principal study variables were consistent with the proposed hypotheses and provided preliminary support for the subsequent serial mediation analysis.

### 3.3. Association Between Bullying Victimization and Depressive Symptoms: The Mediating Roles of Acculturative Stress and Ego-Resiliency

**Figure 2:**
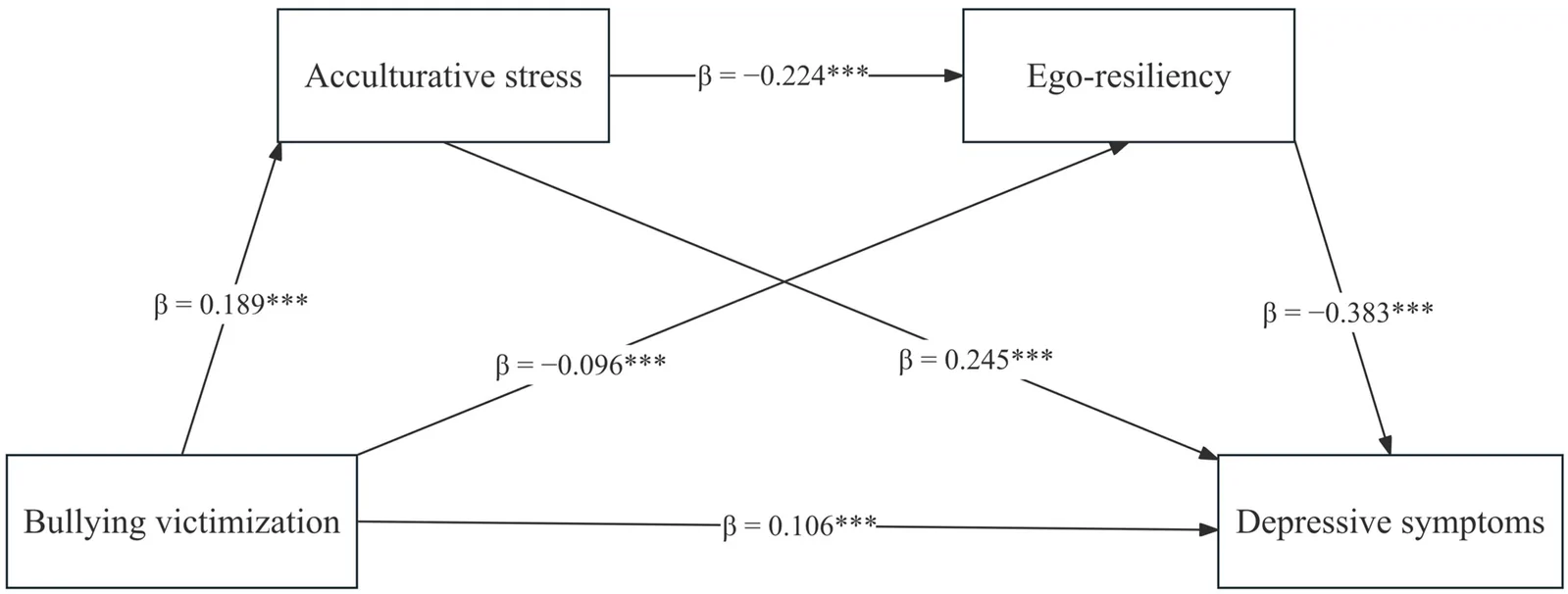
Associations in the theoretically specified serial mediation model of bullying victimization, acculturative stress, ego-resiliency, and depressive symptoms among Korean multicultural adolescents. Standardized path coefficients (β) are presented. *** *p* < 0.001.

As shown in Figure 2, after controlling for sex, age, and perceived household economic status, bullying victimization was significantly and positively associated with acculturative stress (β = 0.189, *p* < 0.001), significantly and negatively predicted ego-resiliency (β = −0.096, *p* < 0.001), and remained significantly and positively associated with depressive symptoms (β = 0.106, *p* < 0.001). Acculturative stress was significantly and negatively associated with ego-resiliency (β = −0.224, *p* < 0.001) and significantly and positively associated with depressive symptoms (β = 0.245, *p* < 0.001). Ego-resiliency, in turn, significantly and negatively predicted depressive symptoms (β = −0.383, *p* < 0.001).

The bootstrap results presented in Table 3 indicated significant indirect associations involving acculturative stress (indirect estimate = 0.187, Boot SE = 0.046, 95% CI [0.104, 0.287]) and ego-resiliency (indirect estimate = 0.149, Boot SE = 0.061, 95% CI [0.037, 0.274]). A significant serial indirect association involving acculturative stress and ego-resiliency was also observed (indirect estimate = 0.066, Boot SE = 0.018, 95% CI [0.036, 0.105]). The 95% bootstrap confidence intervals for all three indirect estimates excluded zero. Collectively, these findings were consistent with the theoretically proposed serial mediation model; however, they do not establish temporal or causal ordering.

The alternative serial indirect association involving ego-resiliency followed by acculturative stress was also statistically significant (indirect estimate = 0.030, Boot SE = 0.010, 95% CI [0.012, 0.051]), indicating that the cross-sectional data could not distinguish between the two mediator orderings.

### 3.4. Network Analysis

#### 3.4.1. Network Structure

**Figure 3:**
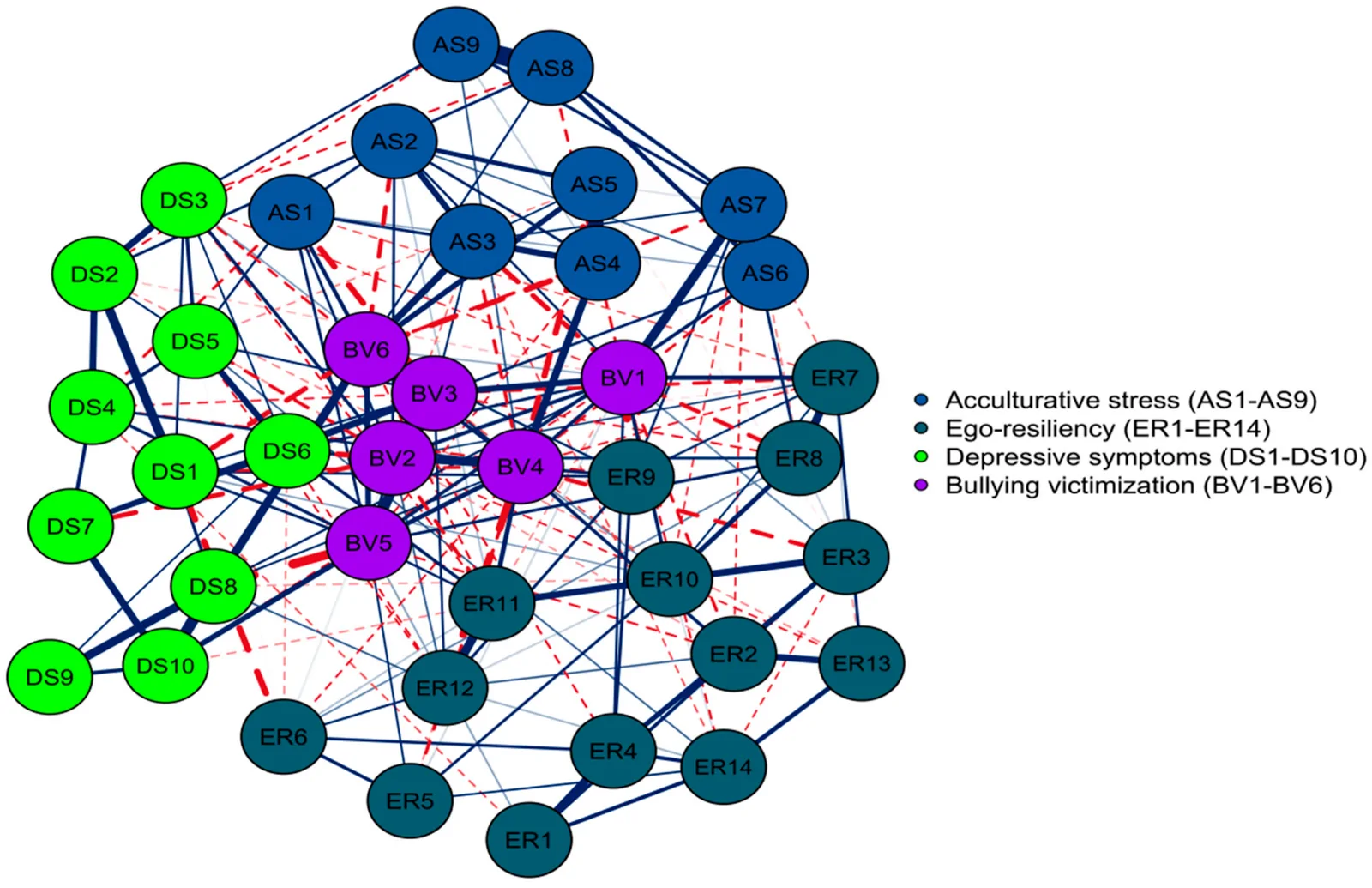
Item-level network of bullying victimization, acculturative stress, ego-resiliency, and depressive symptoms among Korean multicultural adolescents. Blue solid edges indicate positive associations, whereas red dashed edges indicate negative associations. Edge thickness is proportional to the absolute magnitude of the regularized partial correlation, with thicker edges indicating stronger conditional associations. The rings surrounding the nodes represent node predictability.

Figure 3 presents the estimated item-level network of bullying victimization, acculturative stress, ego-resiliency, and depressive symptoms. The network exhibited a relatively dense pattern of connections, with both positive and negative associations observed within and between the four communities. Blue solid lines represent positive associations, whereas red dashed lines represent negative associations. Overall, nodes belonging to the same construct tended to cluster together and showed relatively dense within-community connections. Multiple cross-community connections were also observed among bullying victimization, acculturative stress, ego-resiliency, and depressive symptoms, indicating that these domains were interconnected at the item level. The strongest edges in the overall network were AS8–AS9 (edge weight = 0.803) and AS4–AS5 (edge weight = 0.570), both within the acculturative stress community. Notable positive between-community edges included AS7–BV1 (edge weight = 0.381), DS8–BV6 (edge weight = 0.347), and AS4–BV4 (edge weight = 0.313).

Node predictability estimates are presented in Supplementary Table S1. Predictability values across the 39 nodes ranged from 0.137 to 0.827, with a mean of 0.542, indicating that, on average, 54.2% of the variance in each node was explained by its neighboring nodes in the network. AS9 showed the highest predictability (R^2^ = 0.827), followed by AS8 (R^2^ = 0.824), BV3 (R^2^ = 0.741), AS7 (R^2^ = 0.726), and AS5 (R^2^ = 0.708). AS1 showed the lowest predictability (R^2^ = 0.137), followed by ER9 (R^2^ = 0.230) and ER5 (R^2^ = 0.327). At the community level, acculturative stress nodes had the highest mean predictability (M = 0.614), followed by bullying victimization (M = 0.595), depressive symptoms (M = 0.550), and ego-resiliency (M = 0.467).

#### 3.4.2. Centrality Analysis

Supplementary Figure S1 presents the strength and expected influence of the network nodes. In terms of strength, BV4 (physical aggression), BV6 (appearance-related bullying), and BV1 (peer exclusion) showed the highest values, indicating that these nodes had the strongest overall absolute connections with other nodes in the estimated network. In terms of expected influence, BV5 (rumor spreading), BV3 (social ignoring), and ER10 (openness to new experiences) showed the highest values, indicating that these nodes had the greatest overall signed connectivity within the network.

#### 3.4.3. Bridge Centrality Analysis

**Figure 4:**
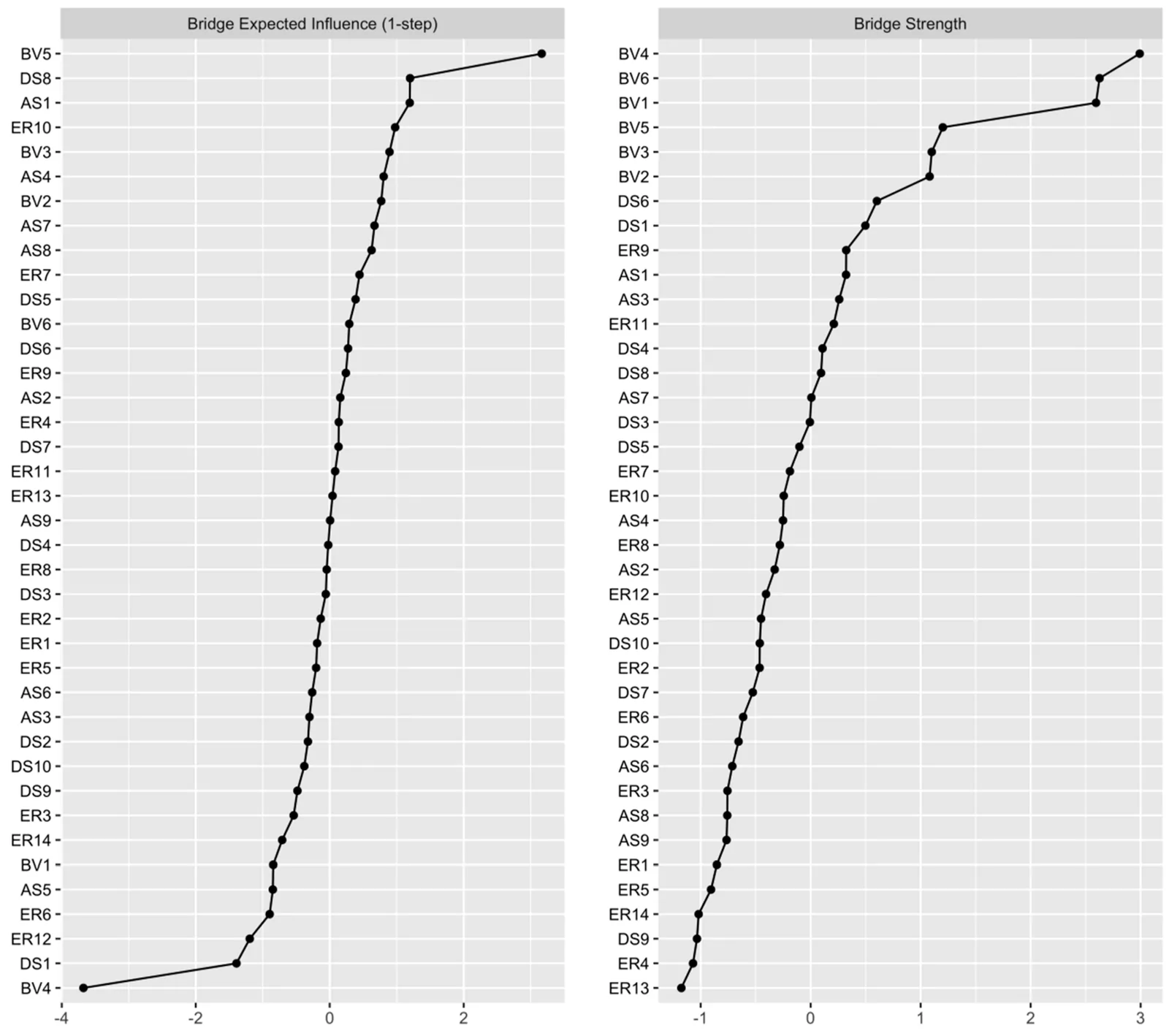
Bridge centrality indices for all nodes in the estimated network. The left panel presents bridge strength, and the right panel presents one-step bridge expected influence. Centrality values are standardized as z scores, with higher values indicating greater bridge centrality. AS = acculturative stress; ER = ego-resiliency; DS = depressive symptoms; BV = bullying victimization.

Figure 4 presents the bridge centrality indices for all nodes in the network. BV5 (rumor spreading) exhibited the highest one-step bridge expected influence, whereas BV4 (physical aggression) and BV6 (appearance-related bullying) showed the highest bridge strength. Overall, several bullying-victimization nodes showed relatively prominent bridge positions and comparatively strong conditional associations with nodes in the other psychological communities.

#### 3.4.4. Network Accuracy and Stability

**Figure 5:**
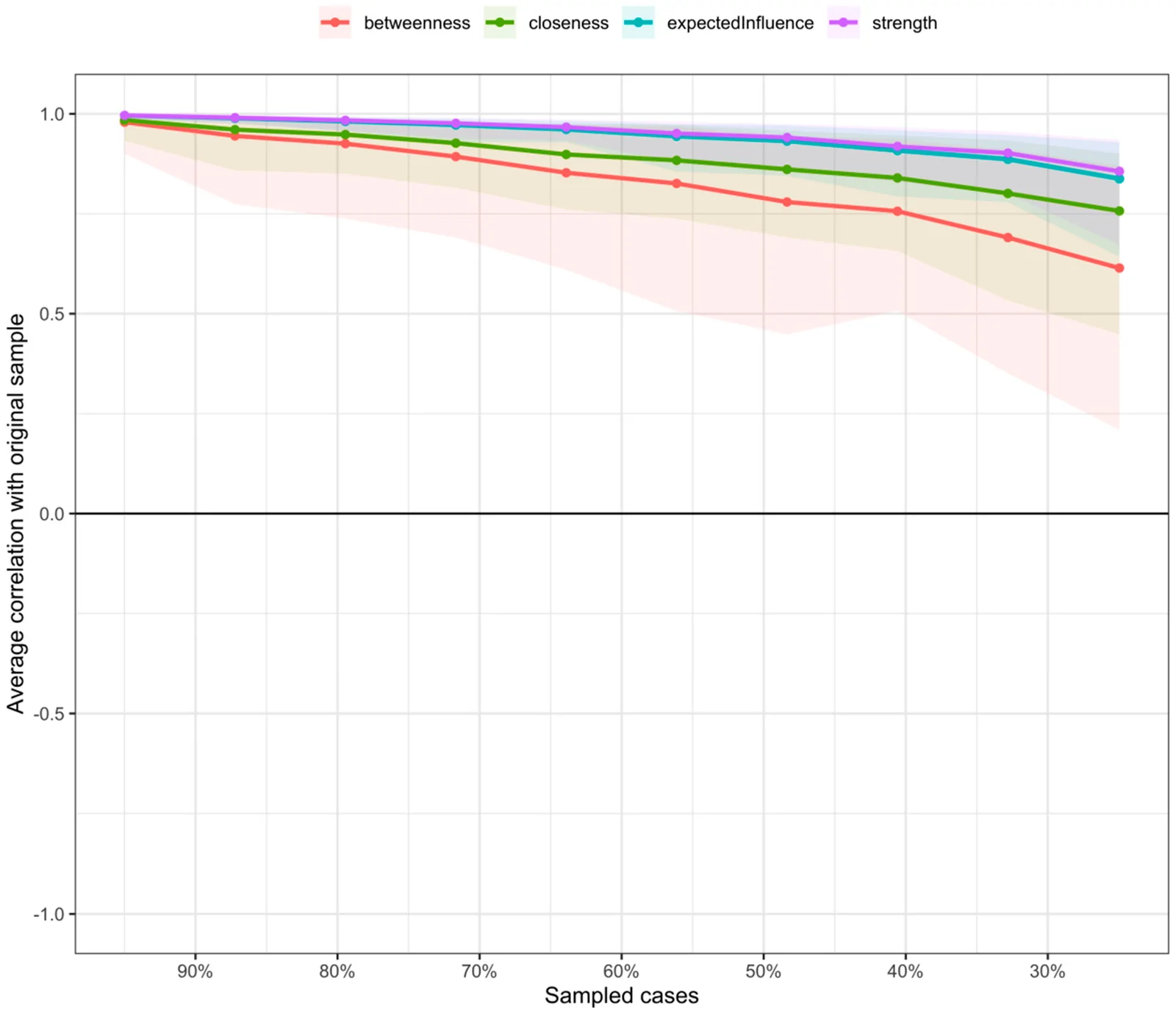
Case-dropping bootstrap analysis of centrality stability. The figure shows the mean correlations between centrality indices estimated from subsets of the data and those estimated from the original sample. Higher correlations indicate greater stability of the corresponding centrality indices.

The accuracy and stability of the estimated network were examined using nonparametric and case-dropping bootstrap procedures, respectively (Figure 5 and Supplementary Figure S2). The mean bootstrapped edge weights were generally close to the corresponding estimates obtained from the original sample, suggesting no substantial systematic bias in the network estimates. The correlation stability coefficients were 0.75 for both strength and expected influence, 0.516 for closeness, and 0.283 for betweenness. Strength and expected influence therefore demonstrated good stability, whereas closeness and particularly betweenness warranted greater caution. Accordingly, the substantive interpretation of node centrality focused on strength and expected influence, and no substantive conclusions were drawn from closeness or betweenness.

## 4. Discussion

The present study integrated construct-level serial mediation and item-level network analysis to examine the interrelationships among bullying victimization, acculturative stress, ego-resiliency, and depressive symptoms in Korean multicultural adolescents. The findings were consistent with a theoretical framework in which sociocultural adversity and individual psychological resources are jointly relevant to adolescent mental health. In particular, the results extend previous research by considering acculturative stress and ego-resiliency together rather than treating them as separate explanatory factors. Nevertheless, because all variables were assessed concurrently, the proposed serial pathway should be understood as a theoretically plausible pattern of associations rather than an established temporal or causal sequence.

The association between bullying victimization and depressive symptoms is consistent with previous evidence linking peer victimization to adolescent emotional difficulties (Hawker & Boulton, 2000; Ye et al., 2023), including depression, suicidal ideation, and other psychological problems (Arseneault, 2018; Klomek et al., 2015; Sahle et al., 2022). According to the interpersonal risk model, experiences of rejection, humiliation, threats, and physical aggression may be associated with a diminished sense of belonging and safety and greater vulnerability to psychological distress (Hammen, 1992). This process may be particularly relevant to multicultural adolescents, whose peer difficulties may be intertwined with cultural background, language proficiency, family origin, and physical appearance (Bahk et al., 2017; Park et al., 2024). Thus, bullying victimization in this population may represent not only interpersonal adversity but also an experience connected to perceived cultural marginalization.

The indirect associations involving acculturative stress and ego-resiliency further illustrate the joint relevance of sociocultural stress and psychological resources. Acculturative stress has been associated with identity conflict, social exclusion, and difficulties adapting to dominant cultural and school environments (Berry, 2005; Ward & Kennedy, 1993), and previous studies have linked it to poorer mental health among Korean multicultural adolescents and other immigrant populations (Bae, 2020; Davydov et al., 2010; González et al., 2001; Mui & Kang, 2006; Son et al., 2024). Ego-resiliency, by contrast, represents a psychological resource associated with adaptation to adversity and lower vulnerability to mental health problems (Davydov et al., 2010; Rutter, 2006). The observed serial indirect association was therefore consistent with the possibility that bullying victimization is related to greater culturally relevant stress, which is in turn associated with fewer resilient resources and more severe depressive symptoms. This interpretation extends studies that examined acculturative stress or resilience separately (Hu et al., 2015; Zhou et al., 2017) and provides a more integrated application of acculturation and resilience perspectives. However, alternative ordering among these variables remains possible and should be evaluated using longitudinal data.

Bullying victimization remained directly associated with depressive symptoms after acculturative stress and ego-resiliency were included in the model, indicating partial statistical mediation. Thus, these two mediators did not account for the entire observed association, and other family-, school-, peer-, and individual-level factors may also be relevant. However, the remaining direct association should not be interpreted as evidence of an unmediated causal effect.

The item-level network analysis complemented the construct-level mediation analysis by demonstrating heterogeneity within the four psychological communities. BV4 (physical aggression), BV6 (appearance-related bullying), and BV1 (peer exclusion) showed relatively high strength values, whereas BV5 (rumor spreading) showed the highest one-step bridge expected influence and BV4 and BV6 showed the highest bridge strength. These patterns suggest that different forms of bullying had distinct conditional associations with the other psychological domains. Acculturative stress nodes, particularly AS8 and AS9, exhibited comparatively high predictability, whereas ego-resiliency nodes showed lower average predictability. Nevertheless, centrality and predictability reflect statistical properties of the estimated network rather than causal or clinical importance and may be affected by item wording, overlapping content, response distributions, and omitted variables (Haslbeck & Fried, 2017; Ning et al., 2025). Accordingly, the network findings should be considered exploratory and should not be interpreted as validating the proposed serial mediation pathway or identifying definitive intervention targets. From a practical perspective, the findings support comprehensive assessment of peer victimization, culturally relevant stress, and psychological resources among multicultural adolescents. School-based support may consider these domains jointly rather than focusing exclusively on depressive symptoms. However, because the present findings are cross-sectional and the network indices do not identify causal intervention targets, the effectiveness of any corresponding prevention or support strategies should be evaluated in longitudinal and intervention studies.

The present study has several strengths. First, it used a large sample from the Multicultural Adolescents Panel Study to examine a population facing distinctive sociocultural challenges. Second, by examining acculturative stress and ego-resiliency within a single serial mediation model, the study integrated risk and protective factors at the construct level. Third, the complementary item-level network analysis characterized heterogeneity in conditional associations that could not be captured using aggregate scores alone. The bootstrap results further supported the stability of the primary centrality indices used for substantive interpretation.

Several limitations should also be acknowledged. First, the cross-sectional design precludes causal inference. Although the observed associations were consistent with the proposed theoretical framework, causal relationships among bullying victimization, acculturative stress, ego-resiliency, and depressive symptoms cannot be established. Longitudinal studies are needed to clarify the temporal ordering and developmental dynamics of these variables. Second, the data were collected in 2015 and may not fully reflect the current experiences of multicultural adolescents in South Korea. Changes in the composition of multicultural families, school environments, public attitudes, and available support services may limit the contemporary relevance of the findings. Future studies using more recent data are therefore needed to determine whether these associations remain applicable in the current social context. Third, because the sample consisted exclusively of multicultural adolescents living in South Korea, the generalizability of the findings to other cultural or ethnic populations may be limited. Future studies should include adolescents from more diverse cultural backgrounds to determine whether the present findings are applicable across different contexts. Fourth, all variables were assessed using self-report questionnaires, which may be subject to reporting bias and common method variance. Future studies incorporating multiple informants or objective assessment methods would strengthen the validity of the findings. Fifth, although age, sex, and perceived family economic status were included as covariates, other potentially relevant factors, such as parental country of origin, place of residence, Korean-language proficiency, family relationships, school adjustment, and social support, were not included in the analytic models. Residual confounding by these factors therefore cannot be excluded. Future studies should incorporate a broader range of family, cultural, school, and social factors to provide a more comprehensive understanding of these associations. Finally, although MAPS was established using a complex sampling design, the serial mediation and network analyses in the present study did not incorporate survey weights, stratification, or clustering. Therefore, the estimated associations and uncertainty measures may not fully reflect the original sampling design, and the findings should be interpreted with caution when generalizing to the broader population of multicultural adolescents in South Korea. Future studies should apply analytic approaches that better accommodate complex survey features when examining these associations.

## 5. Conclusions

The present cross-sectional study found that bullying victimization was associated with depressive symptoms among Korean multicultural adolescents, with significant indirect associations involving greater acculturative stress and lower ego-resiliency, both independently and in the theoretically specified serial order. At the item level, physical aggression, peer exclusion, appearance-related humiliation, and rumor spreading showed relatively high centrality or bridge centrality, whereas acculturative stress related to the perceived mistreatment of one’s family showed particularly high predictability. The serial mediation and network analyses therefore provided complementary construct- and item-level descriptions of the observed associations. However, these findings do not establish temporal or causal ordering, nor do the network indices identify definitive intervention targets. Future longitudinal research is needed to examine the proposed temporal sequence and to evaluate whether school-, cultural-, and resilience-focused interventions are associated with improved mental health among multicultural adolescents.

## Figures and Tables

**Table 1:** Descriptive Characteristics of the Sample.

Variables	N	%	Mean	SD
**Age (years)**			13.97	0.37
**Gender**				
Male	657	49.1		
Female	681	50.9		
**Foreign-born parent status**				
Father is foreign	45	3.4		
Mother is foreign	1290	96.4		
Both parents are foreign	3	0.2		
**Mother’s country of birth**				
Korea	45	3.4		
China (Han or other ethnic groups)	94	7.0		
China (Korean-Chinese)	247	18.5		
Vietnam	34	2.5		
Philippines	340	25.4		
Japan	457	34.2		
Thailand	50	3.7		
Other	71	5.3		
**Perceived household economic status (SES)**				
Very poor	191	14.3		
Poor	543	40.6		
Average	569	42.5		
Good	33	2.5		
Very good	2	0.1		
**Place of residence**				
Large city	354	26.5		
Small city	599	44.8		
Rural area	385	28.8		

**Table 2:** Descriptive statistics and correlations among study variables.

Variables	1	2	3	4	5	6	7
1. Gender	-						
2. Age	−0.015	-					
3. SES	0.004	0.026	-				
4. Bullying victimization	−0.020	0.070 *	−0.019	-			
5. Acculturative stress	−0.019	0.058 *	−0.085 **	0.194 **	-		
6. Ego-resiliency	−0.060 *	0.032	0.048	−0.135 **	−0.241 **	-	
7. Depressive symptoms	0.129 **	0.007	−0.058 *	0.204 **	0.357 **	−0.464 **	-
Mean	1.51	13.97	2.34	6.25	11.81	42.79	16.95
SD	0.500	0.368	0.753	1.325	3.277	6.383	5.358

Note. N = 1388. Values above the mean and SD rows are Pearson correlation coefficients. Sex was coded as 1 = male and 2 = female. SES = perceived household economic status; SD = standard deviation. * *p* < 0.05; ** *p* < 0.01.

**Table 3:** Bootstrap estimates of the total, direct, and indirect effects of bullying victimization on depressive symptoms.

Mediation Pathways	Effect	Boot SE	95% CI	Relative Mediation
Total effect	0.831	0.108	[0.620, 1.042]	-
Direct effect	0.429	0.095	[0.243, 0.615]	51.62%
Total indirect effect	0.402	0.098	[0.222, 0.607]	48.38%
BV → AS → DS	0.187	0.046	[0.104, 0.287]	22.50%
BV → ER → DS	0.149	0.061	[0.037, 0.274]	17.93%
BV → AS → ER → DS	0.066	0.018	[0.036, 0.105]	7.94%

Note. BV, bullying victimization; AS, acculturative stress; ER, ego-resiliency; DS, depressive symptoms; Boot SE, bootstrap standard error; CI, confidence interval. Effects are presented as unstandardized coefficients.

## Data Availability

The data used in this study were obtained from the Multicultural Adolescents Panel Study (MAPS) conducted by the National Youth Policy Institute (NYPI), Republic of Korea. The dataset is publicly available through the NYPI official website (https://www.nypi.re.kr/; accessed on 13 September 2026) in accordance with the institute’s data access policies.

## Supplementary Materials

The following supporting information can be downloaded at: https://www.mdpi.com/article/10.3390/bs16091667/s1.
