# Supplementary material for: How Is Bullying Victimization Associated with Depressive Symptoms Among Multicultural Adolescents in South Korea? A Serial Mediation and Network Analysis of Acculturative Stress and Ego-Resiliency
Source: Behav Sci (Basel). 2026 Sep 16;16(9):1667. doi: 10.3390/bs16091667 (PMC13603447; doi:10.3390/bs16091667)
Supplement: Supplementary file 1 [file behavsci-16-01667-s001.zip › behavsci-4504239-supplementary.pdf]

Supplementary Table S1. Predictability (R^2) of the network nodes

Node	Description	Predictability (R^2)
**Acculturative stress**		
AS1	I feel stressed when other people make fun of the culture of my foreign-born parent.	0.137
AS2	I do not want to go to school because one of my parents is a foreigner.	0.499
AS3	I feel stressed about living in Korea.	0.485
AS4	I feel stressed because I am not good at speaking Korean.	0.679
AS5	People around me pressure me to behave like a Korean.	0.708
AS6	I am looked down upon because one of my parents is a foreigner.	0.645
AS7	My friends exclude me because one of my parents is a foreigner.	0.726
AS8	People in my neighborhood mistreat my family.	0.824
AS9	Korean people mistreat my family.	0.827
**Ego-resiliency**		
ER1	I am tolerant toward my friends.	0.446
ER2	Even when something unexpected happens, I recover quickly and cope with it well.	0.437
ER3	I enjoy trying new things that I have not done before.	0.535
ER4	I tend to make a good impression on other people.	0.556
ER5	I enjoy trying new foods.	0.327
ER6	I am a very energetic person.	0.506
ER7	Even when going to the same place, I prefer taking a different route instead of my usual one.	0.412
ER8	I am more curious than other people.	0.499
ER9	I usually think carefully before I act.	0.230
ER10	I enjoy doing new and different kinds of activities.	0.598
ER11	My daily life is full of interesting things.	0.592
ER12	I can confidently say that I am a strong-willed person.	0.539
ER13	Even when I get angry with someone, I calm down quickly.	0.378
ER14	I like most of the people I meet.	0.478
**Depressive symptoms**		
DS1	I have little energy.	0.574
DS2	I feel unhappy, sad, or depressed.	0.653
DS3	I worry a lot.	0.538
DS4	I have thoughts of wanting to die.	0.467
DS5	I cry easily.	0.392
DS6	When something goes wrong, I often think it is my fault.	0.375
DS7	I feel lonely.	0.565
DS8	I have no interest in or enjoyment of anything.	0.646
DS9	I feel that my future is not hopeful.	0.605

DS10	Everything feels difficult to me.	0.685
**Bullying victimization**		
BV1	I was excluded by other students.	0.512
BV2	I was insulted, severely teased, or mocked by other students.	0.607
BV3	Other students deliberately left me out of activities or completely ignored me.	0.741
BV4	I was hit, kicked, or threatened by other students.	0.497
BV5	Other students spread false rumors about me, causing other friends to dislike me.	0.706
BV6	Other students insulted or mocked me because of my physical characteristics or appearance.	0.506

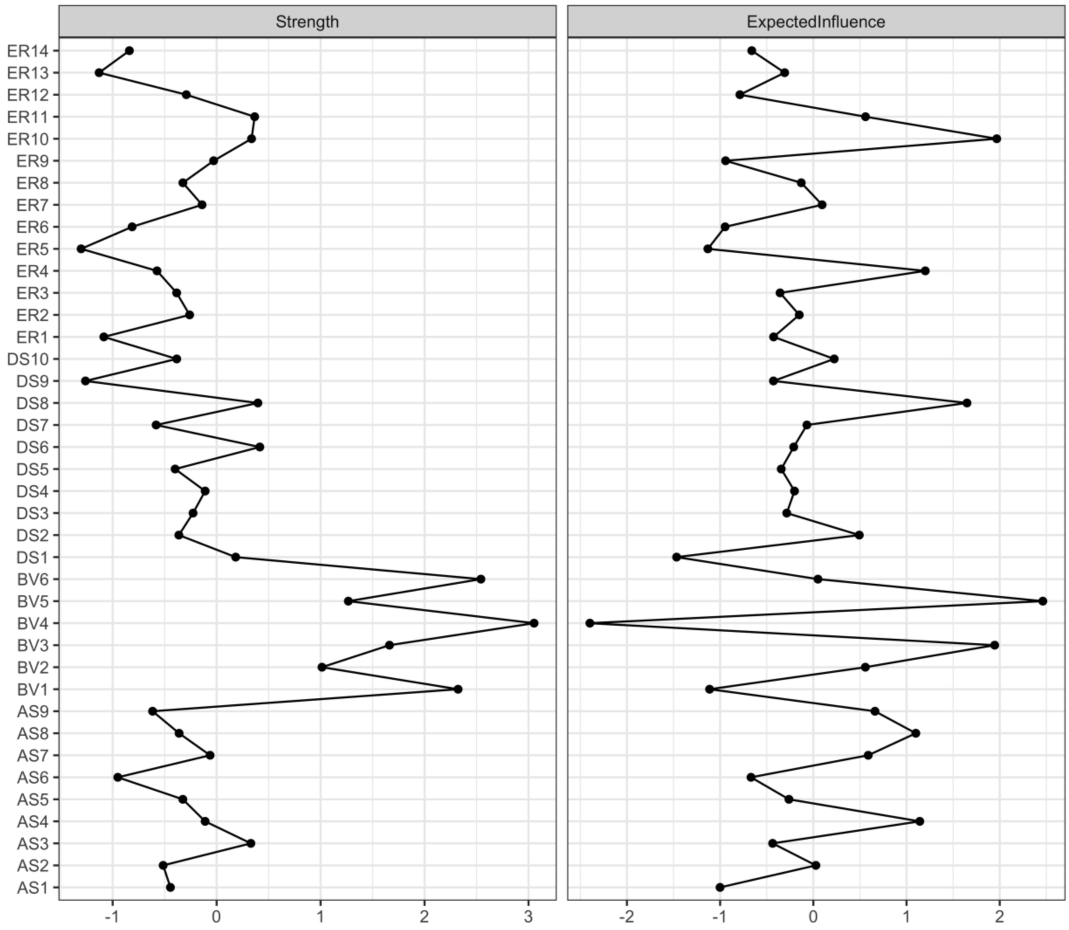

Figure S1. Strength and expected influence of all nodes in the item-level network.

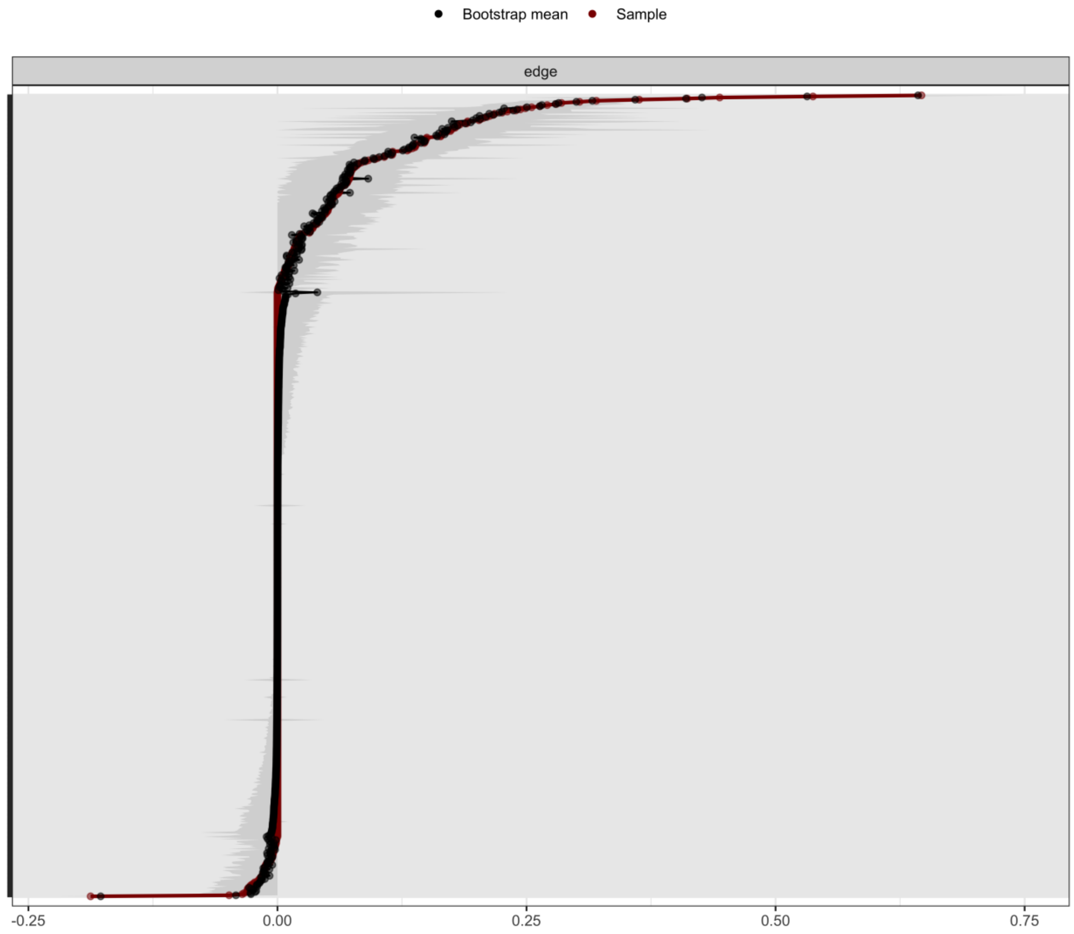

Figure S2. Bootstrapped confidence intervals of the estimated edge weights. The dark red points represent the edge-weight estimates obtained from the original sample, the black points represent the bootstrap means, and the gray bars represent the 95% bootstrap confidence intervals. Wider intervals indicate lower estimation accuracy, whereas narrower intervals indicate greater accuracy.
